# Supplementary material for: Treatment with Testosterone Therapy in Type 2 Diabetic Hypogonadal Adult Males: A Systematic Review and Meta-Analysis
Source: Clin Pract. 2023 Mar 20;13(2):454–69. doi: 10.3390/clinpract13020041 (PMC10037582; doi:10.3390/clinpract13020041)
Supplement: Supplementary file 1 [file clinpract-13-00041-s001.zip › clinpract-2231970-supplementary.pdf]

## Supplementary Table S1: Detailed search strategy

| Database | Search strategy | Results |
| --- | --- | --- |
| **PubMed** | ((((((Testosterone)) OR (Androgen)) OR (androgenic hormone)) AND (((Type 2 diabetes) OR (insulin independent diabetes)) OR (adult-onset diabetes))) AND (treatment)) AND (male hypogonadism) | 300 |
| **Google Scholar** | ((((((Testosterone)) OR (Androgen)) OR (androgenic hormone)) AND (((Type 2 diabetes) OR (insulin independent diabetes)) OR (adult-onset diabetes))) AND (treatment)) AND (male hypogonadism) | 284 |
| **Embase** | ((((((Testosterone)) OR (Androgen)) OR (androgenic hormone)) AND (((Type 2 diabetes) OR (insulin independent diabetes)) OR (adult-onset diabetes))) AND (treatment)) AND (male hypogonadism) | 75 |

## Supplementary Table S2: Characteristics of RCTs

| **Characteristic** | **Dhindsa (2015)** [13] | **Gianatti (2014)** [8] | **Hackett (2014)** [14] | **Jones (2011)** [15] | **Gopal (2010)** [16] | **Heulfelder (2009)** [17] | **Kapoor (2006)** [18] | **Boyonav (2003)** [19] | **Hackett (2018)** [20] | **Khirpun (2018)** [21] | **Groti (2018)** [22] | **Groti (2020)** [5] | **Wittert (2021)** [23] |
|---|---|---|---|---|---|---|---|---|---|---|---|---|---|
| **Study name** | Insulin Resistance and Inflammation in Hypogonadotropic Hypogonadism and Their Reduction After Testosterone Replacement in Men with Type 2 Diabetes | Effect of Testosterone Treatment on Glucose Metabolism in Men with Type 2 Diabetes: A Randomized Controlled Trial | Testosterone Replacement Therapy Improves Metabolic Parameters in Hypogonadal Men with Type 2 Diabetes but Not in Men with Coexisting Depression: The BLAST Study | Testosterone Replacement in Hypogonadal Men with Type 2 Diabetes and/or Metabolic Syndrome (the TIMES2 Study) | Treatment of hypogonadism with testosterone in patients with type 2 diabetes mellitus | Fifty- two—Week Treatment with Diet and Exercise Plus Transdermal Testosterone Reverses the Metabolic Syndrome and Improves Glycaemic Control in Men with Newly Diagnosed Type 2 Diabetes and Subnormal Plasma Testosterone | Testosterone replacement therapy improves insulin resistance, glycaemic control, visceral adiposity and hypercholesterolaemia in hypogonadal men with type 2 diabetes | Long-term testosterone therapy in type 2 diabetes is associated with reduced mortality without improvement in conventional cardiovascular risk factors | Long-term testosterone therapy in type 2 diabetes is associated with reduced mortality without improvement in conventional cardiovascular risk factors | Influence of testosterone substitution on glycaemic control and endothelial markers in men with newly diagnosed functional hypogonadism and type 2 diabetes mellitus: a randomized controlled trial | The impact of testosterone replacement therapy on glycaemic control, vascular function, and components of the metabolic syndrome in obese hypogonadal men with type 2 diabetes | Testosterone treatment longer than 1 year shows more effects on functional hypogonadism and related metabolic, vascular, diabetic and obesity parameters (results of the 2-year clinical trial) | Testosterone treatment to prevent or revert type 2 diabetes in men enrolled in a lifestyle programme (T4DM): a randomised, double-blind, placebo-controlled, 2-year, phase 3b trial |
| **Patients, n** | 94 | 88 | 211 | 220 | 22 | 32 | 27 | 48 | 857 | 80 | 55 | 55 | 1007 |
| **Enrolment** | 2010 | 2009 | 2008 | 2006 | 2006 | 2005 | 2002 | 1998 | 2007 | 2012 | 2014 | 2014 | 2013 |

| | | | | | | | | | | | | | |
|---|---|---|---|---|---|---|---|---|---|---|---|---|---|
| **Initiation Enrolment completion** | 2014 | 2013 | 2012 | 2007 | 2009 | 2008 | 2006 | 2002 | 2009 | 2017 | 2018 | 2018 | 2017 |
| **Year of completion** | 2014 | 2014 | 2014 | 2011 | 2010 | 2009 | 2006 | 2003 | 2018 | 2018 | 2018 | 2020 | 2021 |
| **Population** | Male subjects with type 2 diabetes between the ages of 30 and 65 years, HbA1c #8% (64 mmol/mol), and stable diabetes regimen for 3 months | Study subjects were recruited from specialist diabetes clinics, primary care, and the general community | Patients were recruited from routine diabetes assessment | Male subjects with diabetes and hypogonadism. | Patients with T2DM in the 25- to 50-year age-group who were diagnosed with hypogonadism were included in the study. | Male patients with type 2 diabetes and hypogonadism | men aged over 30 years with type 2 diabetes and with hypogonadism. | middle-aged men with both type 2 diabetes mellitus and mild androgen deficiency. | Male subjects with diabetes and hypogonadism. | men newly diagnosed with T2DM according to the criteria of the American Diabetes Association and referring to HbA1c levels as leading parameter (HbA1c levels had to be measured two times within 4 weeks above 6.5% | Obese males with hypogonadism and diabetes | Obese males with hypogonadism and diabetes | men newly diagnosed with T2DM according to the criteria of the American Diabetes Association and referring to HbA1c levels as leading parameter (HbA1c levels had to be measured two times within 4 weeks above 6.5% |
| **Trial type** | randomized, parallel, placebo controlled, double-blind, prospective, single-centre trial | randomized, double-blind, placebo-controlled trial | double-blind, placebo-controlled intervention study | prospective, randomized, double-blind, placebo-controlled, multicentre study | double-blind, placebo-controlled, crossover study | randomized, double-blind, placebo-controlled trial | double-blind placebo-controlled crossover study | open-label, randomized, no-treatment controlled study | double -blind randomised placebo - controlled study | double -blind randomised placebo - controlled study | double -blind randomised placebo - controlled study | double - blind randomised placebo - controlled study | randomised, double-blind, placebo-controlled |

| | | | | | | | | | | | | | |
|---|---|---|---|---|---|---|---|---|---|---|---|---|---|
| **Inclusion Criteria** | Male subjects with type 2 diabetes between the ages of 30 and 65 years, HbA1c #8% (64 mmol/mol), and stable diabetes regimen for 3 months were recruited between December 2010 and January 2014 | Men aged 35–70 years of age were eligible to participate in this trial if they had a history of T2D, and the total testosterone (TT) level (averaged from two fasting morning specimens) was #12.0 nmol/L (346 ng/dL). | Inclusion criteria for the BLAST intervention study were men aged 18–80 with type 2 diabetes and willing to give written informed consent. Eligibility included an initial finding of either a total testosterone between 8.1 and 12 nmol/L or FT 0.181–0.25 nmol/L (mild group), or total testosterone of 8.0 nmol/L or less or 0.18 nmol/L FT or less (severe group) according to the current 2006 ISSAM EAU guidelines, and with symptoms of | Men aged $40 years were eligible to enter the study if they had confirmed hypogonadism (early morning [08002 1000 h] total testosterone [TT] #11 nmol/L or free testosterone #255 pmol/L on two occasions $1 week apart), with at least two symptoms of hypogonadism (14) and fulfilled criteria for type 2 diabetes (15) and/or MetS | Patients with T2DM in the 25- to 50-year age-group who were diagnosed with hypogonadism were included in the study. Hypogonadism was defined as a calculated free testosterone (cFT) level less than 64.8 pg/mL (0.225 nmol/L) on at least 2 occasions in the presence of symptoms of hypogonadism. | males with the MetS and newly diagnosed T2D (fasting plasma glucose .7.0 at baseline and/or .11.1 after a 2-hour, 75-g oral glucose tolerance test, and an elevated level of HbA1c) | Type 2 diabetic men with HbA1c up to 9.5% showing no significant symptoms of hyperglycaemia. Hypogonadism was defined as total testosterone level!12 nmol/l (on two separate occasions) and symptoms of hypogonadism (positive ADAM score) | subjects had to be aged between 45 and 65 years, be married or living in a stable relationship with a female sexual partner for at least 6 months, have a waist–hip ratio (WHR) of at least 0.9, have symptoms of andropause or erectile dysfunction, and have serum testosterone | Men aged 18 to 80 years with an initial finding of either a TT (on 2 separate occasions) ≤12 nmol/L or FT≤0.18 nmol/L with symptoms of HG defined by the Ageing Male Symptom score. | Men with newly diagnosed diabetes potential functional hypogonadism according to the diagnostic criteria of the EAU guideline on male hypogonadism as of 2015 (serum levels of total testosterone two times below 12.1 nmol/L or serum levels of free testosterone two times below 243 pmol/L in combination of at least two symptoms or complaints of sexual or psychological nature) | men aged > 35 years body mass index > 30 kg/m2 confirmed hypogonadism type 2 diabetes mellitus treated with non-insulin therapy | men aged > 35 years body mass index > 30 kg/m2 confirmed hypogonadism type 2 diabetes mellitus treated with non-insulin therapy | . Men aged 50–74 years, with a waist circumference of 95 cm or higher, a serum testosterone concentration of 14·0 nmol/L or lower but without pathological hypogonadism, and impaired glucose tolerance (oral glucose tolerance test [OGTT] 2-h glucose 7·8–11·0 mmol/L) or newly diagnosed type 2 diabetes (provided OGTT 2-h glucose ≤15·0 mmol/L) |

| | | | | | | | | | | | | | |
|---|---|---|---|---|---|---|---|---|---|---|---|---|---|
| | | | hypogonadism defined by the ageing male symptom (AMS) | | | | | | | | | | |
| **Exclusion criteria** | Subjects on androgens, glucocorticoids, or opiates in the last 6 months or with panhypopituitarism, congenital HH, prolactinoma, head trauma, severe hepatic or kidney disease (glomerular filtration rate ,30 mL/min/m2), HIV, prostate specific antigen .4 ng/mL, or contraindications to testosterone replacement therapy were excluded from the study. | Exclusion criteria included testosterone treatment within 5 years prior to randomization, established pituitary or testicular disorder, screening TT level of ,5.0 nmol/L (144 ng/dL), luteinizing hormone (LH) level .1.53 upper limit of normal, or screening prostate-specific antigen (PSA) level. 4 mg/L, a history of urinary obstruction, prostate cancer, or breast cancer, hematocrit .0.50, uncontrolled hypertension (.160/90 mmHg despite treatment), untreated obstructive | Exclusion criteria included any past history of testosterone replacement, history of prostate, breast or hepatic cancer, abnormal digital rectal examination, severe symptoms of prostate hypertrophy or elevated prostate-specific antigen (PSA) (>4 mm// L), or hematocrit of 55% or greater. If, in the opinion of the principal investigator, the cause of elevated PSA was well-controlled | Exclusion criteria included TRT within 6 months of randomization, hormone modulating therapies or topical/systemic glucocorticoids within 3 months of randomization, or insulin therapy; a history of/ current prostate or breast cancer; abnormal digital rectal examination suggestive of prostate carcinoma; severe symptomatic benign prostatic hyperplasia or | Those with a history of pre-existing hypogonadism, panhypopituitarism, chronic renal failure, chronic liver disease, chronic alcoholism, radiation therapy, inflammatory disease or active infection, hormone therapy, or any contraindication to testosterone therapy such as elevation of the prostate-specific antigen (PSA) value beyond the age-adjusted reference | - | Patients were excluded if they had any inflammatory disease or infection with elevation of C-reactive protein O10 mg/l, were already on hormone therapy or had any contraindication to testosterone therapy such as elevation of prostate-specific antigen (PSA) beyond the age-adjusted normal range. | Subjects were excluded from the trial if they had concurrent illnesses other than diabetes or surgical interventions likely to impair sexual function, severe diabetic complications such as amputations or chronic renal failure, drug use other than antidiabetic medication or antihyper-tensives (angiotensin-converting enzyme (ACE inhibitors), history of alcoholism or major psycho-pathology; or any sign or | Exclusion criteria included any past history of testosterone therapy, history of prostate, breast or hepatic cancer, abnormal digital rectal examination, severe symptoms of prostate hypertrophy or elevated prostate-specific antigen>4 ng/mL or haematocrit≥55%. | Exclusion criteria were primary or secondary hypogonadism of any origin (testicular damage, diagnosis of pituitary/hypothalamic malfunction), as well as hyperprolactinemia and hypothyroidism. Also, any history of a malignant disease as well as elevated levels of PSA (>4 mg/dL) and the wish for paternity led to exclusion. | previously treated hypogonadism the 2-diabetes mellitus treated with insulin therapy a history of current prostate or breast cancer severe benign prostatic hyperplasia elevated prostate-specific antigen (PSA | previously treated hypogonadism the 2-diabetes mellitus treated with insulin therapy a history of current prostate or breast cancer severe benign prostatic hyperplasia elevated prostate-specific antigen (PSA > 4.0 lg/l) severe heart failure acute coronary event or procedure during the six months leading up to the study | p rimary or secondary hypogonadism of any origin (testicular damage, diagnosis of pituitary /hypothalamic malfunction), as well as hyperprolactinemia and hypothyroidism. Also, any history of a malignant disease as well as elevated levels of PSA (>4 mg/dL) and the wish for paternity led to exclusion. |

| | | | | | | | | | | | | | |
|---|---|---|---|---|---|---|---|---|---|---|---|---|---|
| | | sleep apnea, estimated glomerular filtration rate , 30 mL/min, cardiac insufficiency (New York Heart Association score .2), active malignancy, unstable psychiatric disease, weight .135 kg (the weight limit for the dual-energy X-ray absorptiometry [DXA] scanner), current use of glucagonlike peptide-1 agonist therapy or very low-calorie diet, or an | BPH, and malignancy had been recently excluded, patients were eligible | elevated age-specific prostate-specific antigen (PSA). | range; patients with an American Urological Association questionnaire (used for symptoms of prostatism) score >22; and those with uncontrolled blood glucose levels—hemoglobin A1c (A1C) >10% | | | evidence of prostate enlargement or abnormalities. | | | > 4.0 lg/l) severe heart failure acute coronary event or procedure during the six months leading up to the study chronic obstructive lung disease hypothyroidism severe obstructive sleep apnea (OSA) active infection rheumatoid arthritis | chronic obstructive lung disease hypothyroidism severe obstructive sleep apnea (OSA) active infection rheumatoid arthritis | |
| **Treatments** | 250 mg testosterone cypionate (Watson | Intramuscular testosterone undecanoate 1,000 mg or a | Subjects were randomized to receive | Subjects were randomized (1:1) to | Testosterone cypionate (Cernos), | Patients were randomized to either supervised diet | Sustanon 200 mg (testosterone propionate | oral testosterone undecanoate | long-acting testosterone undecanoate | T-Gel at a dose of 50 mg per day | testosterone undecanoate | testosterone undecanoate 1000 | intramuscular injection of testoster |

| | | | | | | | | | | | | |
|---|---|---|---|---|---|---|---|---|---|---|---|---|
| | Pharmaceuticals, New Jersey; 200 mg/mL) or placebo (1.25 cc saline) intramuscularly in the buttock. | visually identical placebo injection (both in oily base) was injected into the upper outer quadrant of the buttock at 0, 6, 18, and 30 weeks | either TU 1,000 mg at week 0, week 6, week 18, administered by the practice nurse or GP over 5 minutes into the right or left upper outer buttock, or matching placebo | receive either 3 g metered-dose 2% testosterone gel (60 mg testosterone, Tostran [also known as Fortigel, Tostrex, Itnogen, Foresta; ProStrakan, Galashiels, Scotland, U.K.]) or placebo gel once daily | 200 mg, a depot preparation of testosterone administered by deep intramuscular injection. Placebo was given as 0.9% isotonic saline | and exercise (D&E) alone or in combination with testosterone gel (50 mg once daily; Testo gel; Bayer Schering Pharma AG, Berlin, Germany) | 30 mg, testosterone phenylpropionate 60 mg, testosterone isocaproate 60 mg, and testosterone decanoate 100 mg/ml, Organon Laboratories, Cambridge, UK), a depot preparation. | (TU; AndriolÒ, Organon, Oss, The Netherlands) for 3 months, at a daily oral dosage of 120 mg, divided into 80 mg at breakfast and 40 mg at dinner (during the meals). | | 1000 mg intramuscular injections two years; according to the protocol every 10 weeks. Placebo arm patients were receiving placebo throughout the first year of this study and testosterone undecanoate 1000 mg intramuscular injections during second year. | mg intramuscular injections two years; according to the protocol every 10 weeks. Placebo arm patients were receiving placebo throughout the first year of this study and testosterone undecanoate 1000 mg intramuscular injections during second year. | one undecanoate (1000 mg) or placebo at baseline, 6 weeks, and then every 3 months for 2 years. |

| | | | | | | | | | | | | | | |
|---|---|---|---|---|---|---|---|---|---|---|---|---|---|---|
| **Primary Outcomes** | The impact of HH on insulin resistance, inflammation, and body composition in men with type 2 diabetes | The primary outcome measure was the change across groups and time from baseline in the homeostasis model assessment index of insulin resistance (HOMA-IR). | The primary outcome measure was the change across groups and time from baseline in the homeostasis model assessment index of insulin resistance (HOMA-IR). | The primary end point was the difference between treatment groups in homeostasis model assessment (HOMA)-IR from baseline to months 6 and 12. | changes in the homeostasis model assessment (HOMA) index of IR, fasting blood glucose level, and A1C value. | The primary end point was the difference between treatment groups in homeostasis model assessment (HOMA)-IR from baseline to months 6 and 12 | Changes in the homeostasis model assessment (HOMA) index of insulin resistance, fasting blood glucose and glycated haemoglobin. | changes in the homeostasis model assessment (HOMA) index of IR, fasting blood glucose level, and A1C value. | The primary outcome measure was the change across groups and time from baseline in the homeostasis model assessment index of insulin resistance (HOMA-IR). | The primary outcome measure was the change across groups and time from baseline in the homeostasis model assessment index of insulin resistance (HOMA-IR). | Effects of testosterone replacement therapy on glycaemic control - fasting plasma glucose (FPG) mmol/l, HbA1c, HOMA-IR, vascular function - change in flow mediated dilatation (FMD) %. vascular morphology - intima-media thickness (IMT) | Effects of testosterone replacement therapy on glycaemic control - fasting plasma glucose (FPG) mmol/l, HbA1c, HOMA-IR, vascular function - change in flow mediated dilatation (FMD) %. vascular morphology - intima-media thickness (IMT) | type 2 diabetes (2-h OGTT glucose ≥11·1 mmol/L) and mean change from baseline in 2-h OGTT glucose, assessed by intention to treat. |

| | | | | | | | | | | | | | |
|---|---|---|---|---|---|---|---|---|---|---|---|---|---|
| **Secondary outcomes** | - | The secondary outcome measure was the change across group and time in glycaemic control as measured by HbA1c. Other outcome measures were considered as explanatory variables. | The secondary outcome measure was the change across group and time in glycaemic control as measured by HbA1c. Other outcome measures were considered as explanatory variables. | Secondary end points were changes from baseline in HbA1c, fasting insulin, FPG, lipid parameters, body composition, BMI, waist circumference, AMS and IIEF scores, AEs, and other safety parameters. HOMA of b-cell function (HOMA-B) was determined post hoc | changes in fasting lipids, anthropometric factors including BMI, waist circumference, and WHR, blood pressure, and androgen deficiency symptoms | Secondary end points were changes from baseline in HbA1c, fasting insulin, FPG, lipid parameters, body composition, BMI, waist circumference, AMS and IIEF scores, AEs, and other safety parameters. HOMA of b-cell function (HOMA-B) was determined post hoc | Changes in fasting lipids, blood pressure and anthropometric measurements including waist circumference, waist/ hip ratio, BMI and % body fat | changes in fasting lipids, anthropometric factors including BMI, waist circumference, and WHR, blood pressure, and androgen deficiency symptoms | Secondary end points were changes from baseline in HbA1c, fasting insulin, FPG, lipid parameters, body composition, BMI, waist circumference, AMS and IIEF scores, AEs, and other safety parameters. HOMA of b-cell function (HOMA-B) was determined post hoc | Secondary end points were changes from baseline in HbA1c, fasting insulin, FPG, lipid parameters, body composition, BMI, waist circumference, AMS and IIEF scores, AEs, and other safety parameters. HOMA of b-cell function (HOMA-B) was determined post hoc | Effects of testosterone replacement therapy on non-alcoholic fatty liver disease (NAFLD), bone mineral density (BMD), total testosterone (TT), prostate specific antigen and haematocrit | Effects of testosterone replacement therapy on non-alcoholic fatty liver disease (NAFLD), bone mineral density (BMD), total testosterone (TT), prostate specific antigen and haematocrit | monitoring of haematocrit and prostate-specific antigen, and analysed prespecified serious adverse events |
| **Follow up** | 24 weeks | 40 weeks | 52 weeks | 12 months | 7 months | 52 weeks | 7 months | 3 months | 3.4 years | 9 months | 1 year | 2 years | 2 years |

## Supplementary Table S3: Characteristics of Observational Studies

| **Characteristics** | **Yassin (2019)** [12] | **Haider (2020)** [24] |
|---|---|---|
| Study name | Testosterone Therapy in Men with Hypogonadism Prevents Progression from Prediabetes to Type 2 Diabetes: Eight-Year Data from a Registry Study | Remission of type 2 diabetes following long-term treatment with injectable testosterone undecanoate in patients with hypogonadism and type 2 diabetes: 11-year data from a real-world registry study |
| Patients, n | 316 | 356 |
| Initiation | 2011 | 2008 |
| Completion | 2018 | 2019 |
| Year of publication | 2019 | 2020 |
| Population | Patients in this study were pooled from two ongoing urological registries. Ethical guidelines by the German Medical Association for observational studies in patients receiving standard treatment were followed | Patients with diabetes managed by the same local diabetes centre |
| Inclusion criteria | Prediabetes, defined as HbA1c 5.7–6.4% (39–46 mmol/mol), and total testosterone levels #12.1 nmol/L (;350 ng/dL) combined with symptoms of hypogonadism. | Patients with T2DM who had total testosterone levels ≤12.1 nmol/L (350 ng/dL) and symptoms of hypogonadism |
| Exclusion criteria | -- | ... |
| Primary Outcome | Anthropometric and metabolic parameters | glucose intolerance, with glycated haemoglobin (HbA1c) and insulin secretion |

## Supplementary Table S4: New Castle Ottawa scale to assess Publication bias in Observational studies

| Study | Selection | | | | Comparability | Outcomes | | | Total |
|---|---|---|---|---|---|---|---|---|---|
| | Representativeness of the Exposed Cohort | Selection of the Non-Exposed Cohort | Ascertainment of Exposure | Demonstration That Outcome of Interest Was Not Present at Start of Study | Comparability of Cohorts on the Basis of the Design or Analysis | Assessment of Outcome | Was Follow-Up Long Enough for Outcomes to Occur | Adequacy of Follow Up of Cohorts | |
| **Yassin (2019)** [12] | * | * | * | * | ** | * | * | * | ********* |
| **Haider (2020)** [24] | * | * | * | * | * | * | * | * | ******** |

**Supplementary Table S5: Cochrane risk of bias tool for assessing publication bias in Randomized controlled trials**

| | Random sequence generation (selection bias) | Allocation concealment (selection bias) | Blinding of participants and personnel (performance bias) | Blinding of outcome assessment (detection bias) | Incomplete outcome data (attrition bias) | Selective reporting (reporting bias) | Other bias |
|---|---|---|---|---|---|---|---|
| Boyanov 2003 [19] | + | + | + | + | − | + | + |
| Dhindsa 2015 [13] | + | − | + | + | + | + | − |
| Gianatti 2014 [8] | − | − | + | + | + | + | + |
| Gopal 2010 [16] | + | − | + | + | + | − | + |
| Groti 2018 [22] | + | + | + | + | − | + | − |
| Groti 2020 [5] | + | + | − | + | + | − | + |
| Hackett 2014 [14] | − | + | + | − | − | + | + |
| Hackett 2018 [20] | + | + | + | + | − | + | + |
| Heufelder 2009 [17] | + | + | + | + | − | + | + |
| Jones 2011 [15] | + | + | + | + | + | + | − |
| Kapoor 2006 [18] | + | − | + | + | + | + | − |
| Khirpun 2018 [21] | + | − | + | + | + | + | − |
| Wittert 2021 [23] | − | + | + | + | + | + | − |

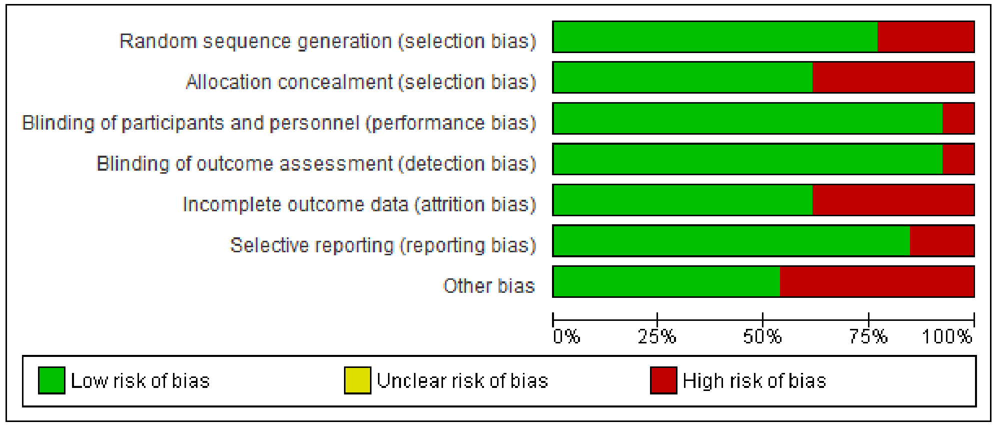

# Supplementary Figure S1: Funnel Plots of primary outcomes

A

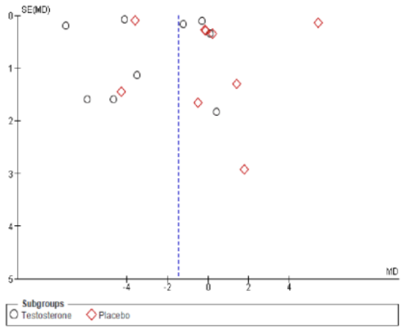


B

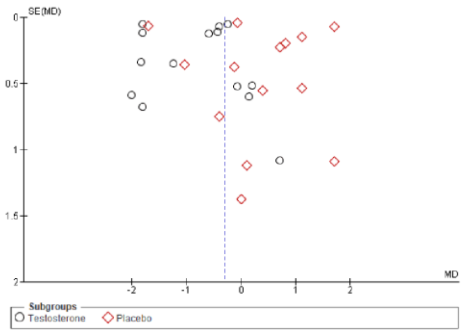


C

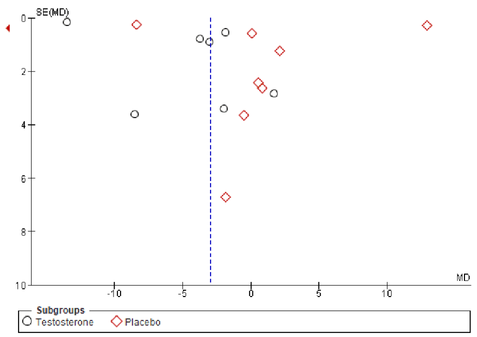


D

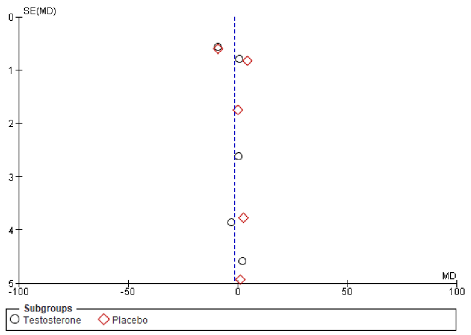

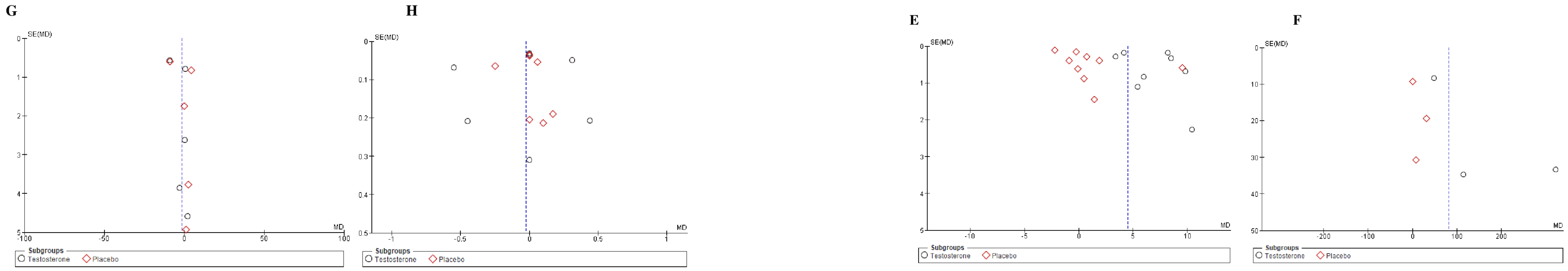


A: Homeostatic model assessment for insulin resistance (HOMA-IR), B: Fasting plasma glucose (FPG), C: Fasting serum insulin (FSI), D: Glycated hemoglobin (HbA1C), E: Total testosterone (TT), F: Free testosterone (FT), G: Sex hormone binding globulin (SHBG), H: Prostate specific antigen (PSA)
